# Supplementary material for: Ablation provides key macronutrients (nitrogen and phosphorous) to glacier ice algae in NW Greenland
Source: Nat Commun. 2026 Jan 28;17:2129. doi: 10.1038/s41467-026-68625-8 (PMC12957424; doi:10.1038/s41467-026-68625-8)
Supplement: Supplementary file 1 — Supplementary Information [file 41467_2026_68625_MOESM1_ESM.pdf]

Supplementary Information

Calculations - Cell lysis estimates.

We estimated how much DOP and DON could have been derived from cell lysis using the average cell counts at each site (Supplementary Table S1). In order to do this, we used the mass of C per cell (106 pg C cell^{-1} [1]) and estimated the moles of C stored within cells at each site. Then, using the average cell stochiometric ratios for C:N and C:P, 19:1 and 509:1 respectively[2], we estimated the N and P stored within the measured cells per ml of melted ice. Finally, we divide the concentrations of DON and DOP by the estimated moles of N and P stored within the cells, and multiplied by 100 to convert into a percentage. Thus, lysing roughly 2.2 – 2.8% of cells would produce the concentrations of DON and DOP measured in the Qaanaaq weathering crust and lysing approximately 3.5 – 3.8% of cells would produce the concentrations of DON and DOP measured in the weathering crust of the GrIS.

Calculations - Unweathered ice melt equivalent to N and P in biomass

We estimated how much unweathered ice ablation is required to provide the mass of N and P stored within glacier ice algal cells (using the average cell count measured at each site). In order to do this, we first estimated how much N and P is stored with the algae at each site, we assume that the average *Ancylonema Nordenskiöldii* cell contains 8.8 pM C cell^{-1} [1]. Hence, using equation 1, the mass of C in the glacier ice algal cells is 1.8×10^6 pmol C/ml in Qaanaaq and 7.1×10^5 pmol C/ml in GrIS samples (Supplementary Table S1). Using the median intra-cellular molar C:N and C:P ratios of the glacier ice algal cells, as above [2], we estimated the mass of N and P stored in the glacier ice algae (equation 2 and 3) as 9.3×10^4 pmol N/ml and 3.5×10^3 pmol P/ml at Qaanaaq, and 3.7×10^4 pmol N/ml and 1.4×10^3 pmol P/ml at GrIS (Supplementary Table S1). Finally, the ablation in units of cm, derived from ml (or cm^3) of melt per cm^2 of ice surface, required to give rise to these algal N and P masses from the DIN and SRP held in deeper meteoric ice was obtained by simply dividing the mass of algal N or P by the average DIN and SRP of the deeper ice (Supplementary Table S1) or the average TDN and TDP of the UW ice to account for organic fractions (Supplementary Table S1). The ablation of water was converted into ablation of ice by dividing by the ice density 0.91 g/cm^3, assuming that 1g of unweathered ice melt has a volume of 1 cm^{3}cm^{-3} [3] (equation 4).

$$pmol\ C_{cells}\, mL^{-1} = 8.8\, pmol\ C\ cell^{-1} \times cells\ mL^{-1} \quad (1)$$

$$pmol\ N_{cells}\ mL^{-1} = \frac{pmol\ C_{cells}\ mL^{-1}}{ratio\ C{:}N} \quad (2)$$

$$pmol\ P_{cells}\ mL^{-1} = \frac{pmol\ C_{cells}\ mL^{-1}}{ratio\ C{:}P} \quad (3)$$

$$cm\ ablation = \frac{\left(\frac{pmol\ N_{cells}\ mL^{-1}}{pmol\ N_{ice}\ mL^{-1}}\right)}{0.91\ g\ cm^{-3}}\ or\ \frac{\left(\frac{pmol\ P_{cells}\ mL^{-1}}{pmol\ P_{ice}\ mL^{-1}}\right)}{0.91\ g\ cm^{-3}} \quad (4)$$

Supplementary Table S1 – Cell counts, and estimated mass of C, N and P in the ice surface samples and melt that would equate moles of N and P at the surface.

	**Qaanaaq**	**GrIS**
mass of C per cell (pg/cell)	106	
mol C per cell (pmol/cell)	8.8	
Average cell count (cell/ml)	2.4×10^{5}	0.75×10^{5}
C in cells (pmol/ml)	1.8×10^{6}	7.1×10^{5}
C:N	19	
C:P	509	
N in cells (pmol/ml)	92974	37190
P in cells (pmol/ml)	3471	1388
**Inorganic fraction:**		
UW DIN (pmol/ml)	3000	4300
UW SRP (pmol/ml)	18	20
ml of melt equivalent to N	31	8.6
ml of melt equivalent to P	193	69
cm ablation for N (density of 0.91 g/cm^3)	34	9.5
cm ablation for P (density of 0.91 g/cm^3)	212	76
**Total nutrients:**		
UW TN (pmol/ml)	3.2	4.0
UW TP (pmol/ml)	36	55
ml of melt equivalent to N	29	9.3
ml of melt equivalent to P	96	25
cm ablation for N (density of 0.91 g/cm^3)	32	10
cm ablation for P (density of 0.91 g/cm^3)	105	27

Supplementary Figures

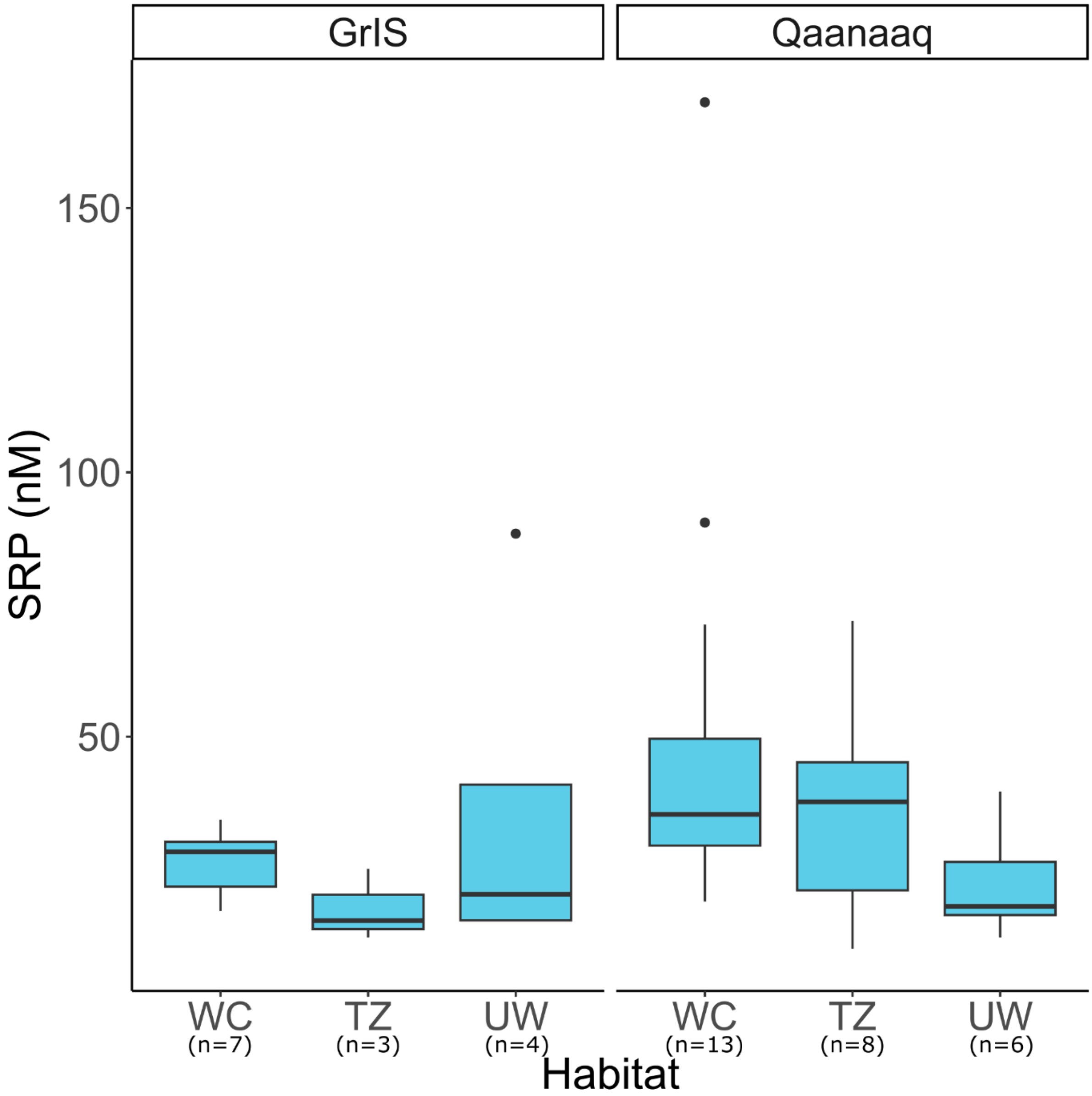

Figure S1. Boxplots showing the concentration of soluble reactive phosphorous (SRP) for weathering crust (WC), transition zone (TZ) and unweathered ice (UW) samples collected at the Qaanaaq sampling locations and Greenland Ice Sheet (GrIS) sampling location. The number off replicates is indicated for each sample type in brackets. The line in the box denotes the median value, the box contains the 25th to 75th percentiles of dataset. The black whiskers mark the 5th and 95th percentiles, and values beyond these upper and lower bounds are considered outliers, marked with dots. No significant differences were found between habitats (Kruskal-Wallis test: $p > 0.05$).

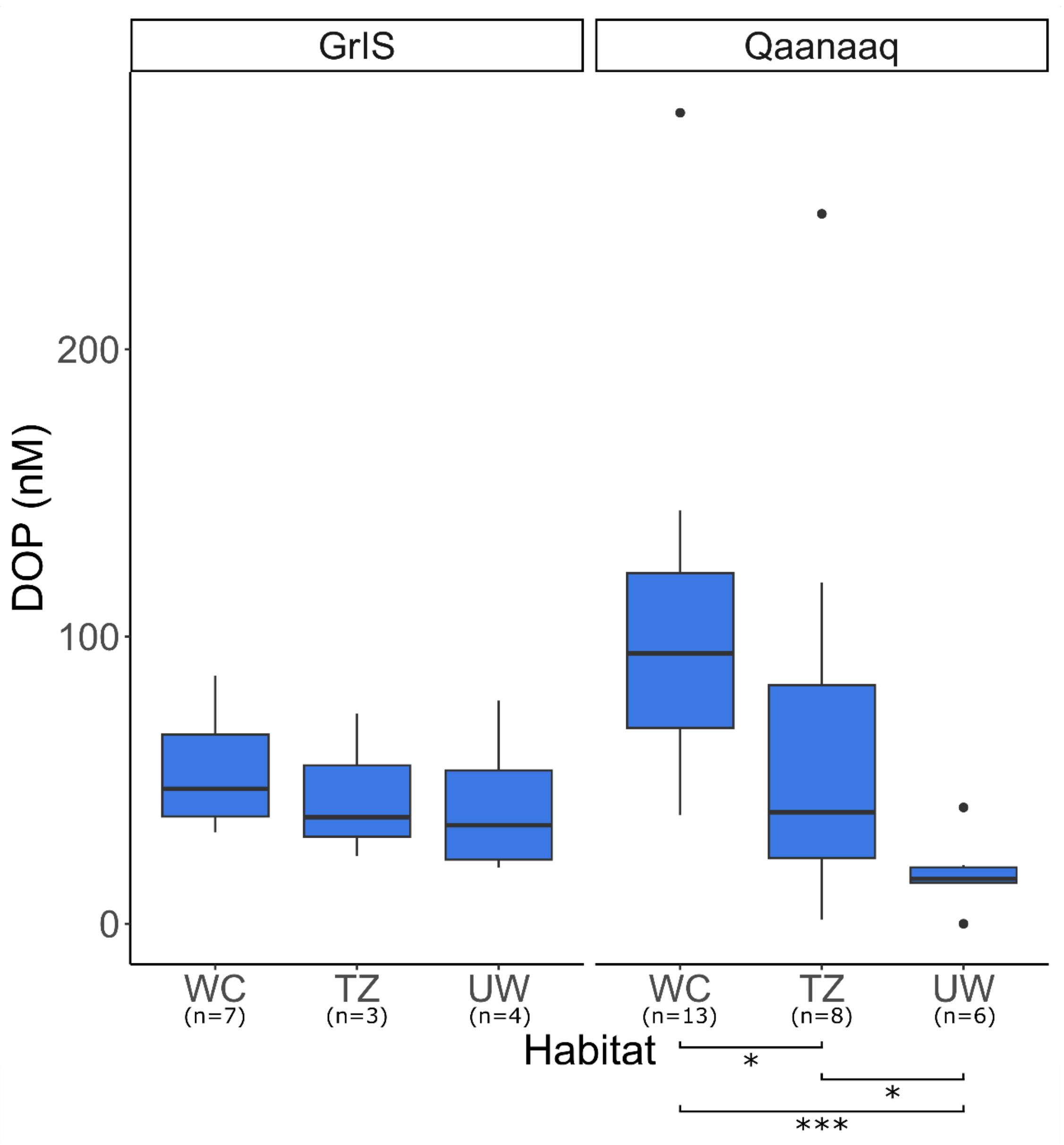

Figure S2. Boxplots showing the concentration of dissolved organic phosphorous (DOP) for weathering crust (WC), transition zone (TZ) and unweathered ice (UW) samples collected at the Qaanaaq sampling locations and Greenland Ice Sheet (GrIS) sampling location. The number off replicates is indicated for each sample type in brackets. The line in the box denotes the median value, the box contains the 25th to 75th percentiles of dataset. The black whiskers mark the 5th and 95th percentiles, and values beyond these upper and lower bounds are considered outliers, marked with dots. Statistical differences between depths, if present, as inferred from a Kruskal-Wallis test and post-hoc Dunns test are noted by “ж” (Dunns test, $p<0.05$), “ж ж” (Dunns test, $p<0.01$) or “ж ж ж” (Dunns test, $p<0.001$).

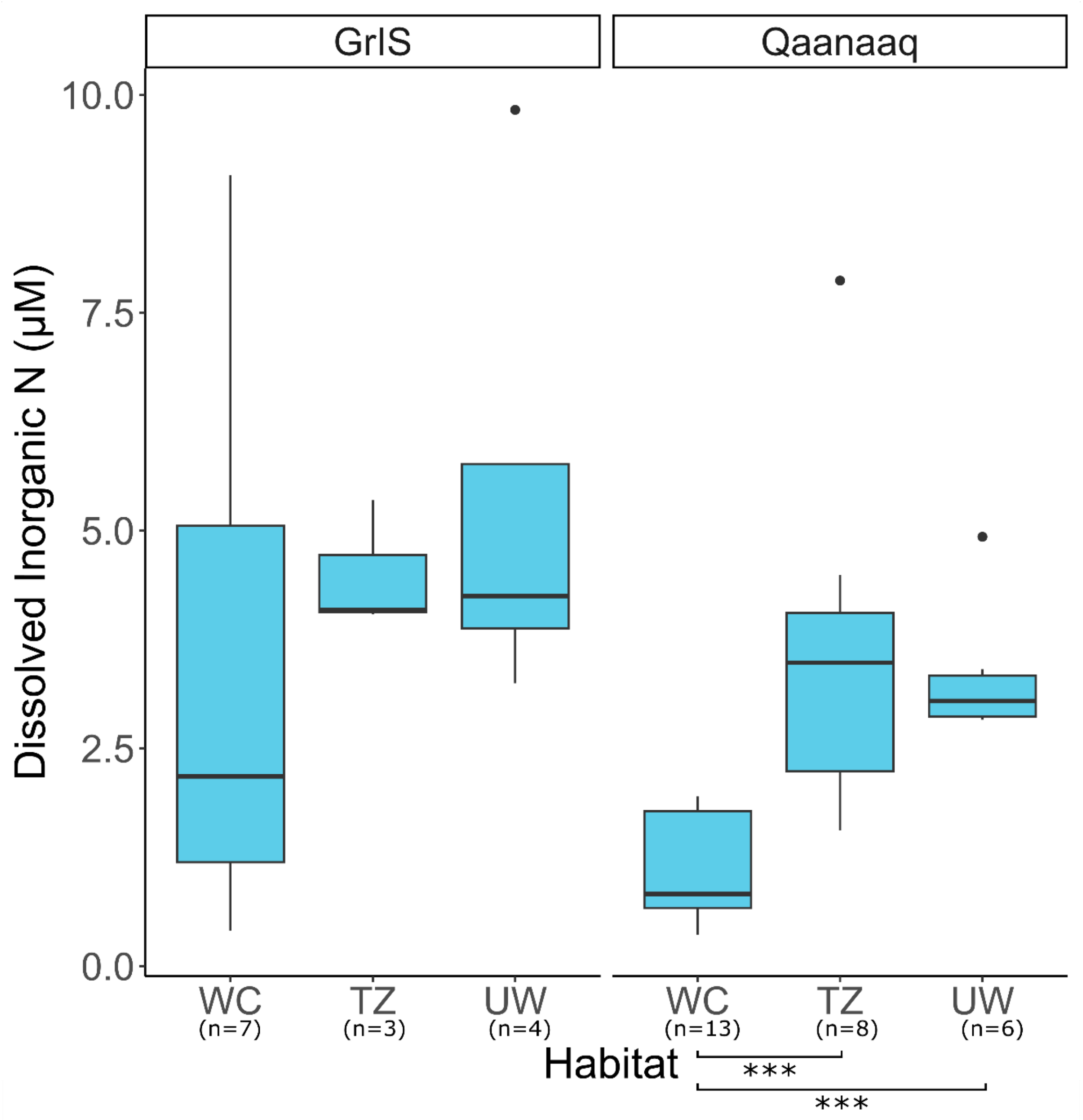

Figure S3. Boxplots showing the concentration of dissolved inorganic nitrogen species (including NH_4^+, NO_3^- and NO_2^-) within weathering crust (WC), transition zone (TZ) and unweathered ice (UW) samples collected at the Qaanaaq sampling locations and Greenland Ice Sheet (GrIS) sampling location. The number off replicates is indicated for each sample type in brackets. The line in the box denotes the median value, the box contains the 25th to 75th percentiles of dataset. The black whiskers mark the 5th and 95th percentiles, and values beyond these upper and lower bounds are considered outliers, marked with dots. Statistical differences between depths, if present, as inferred from a Kruskal-Wallis test and post-hoc Dunns test are noted by "ж" (Dunns test, $p<0.05$), "ж ж" (Dunns test, $p<0.01$) or "ж ж ж" (Dunns test, $p<0.001$).

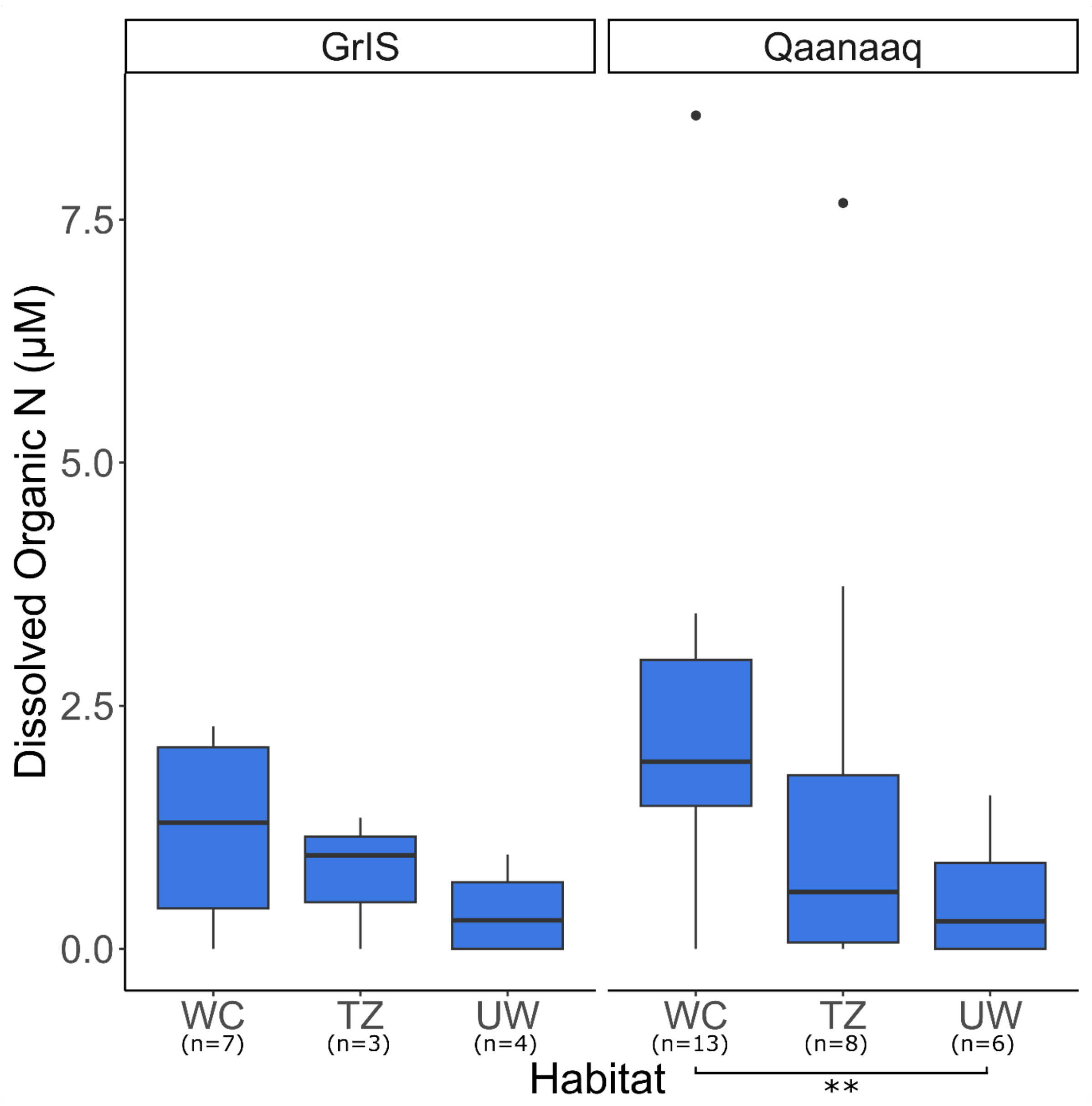

Figure S4. Boxplots showing the concentration of dissolved organic nitrogen species (as determined by the difference between Total Dissolved Nitrogen and DIN) within weathering crust (WC), transition zone (TZ) and unweathered ice (UW) samples collected at the Qaanaaq sampling locations and Greenland Ice Sheet (GrIS) sampling location. The number off replicates is indicated for each sample type in brackets. The line in the box denotes the median value, the box contains the 25th to 75th percentiles of dataset. The black whiskers mark the 5th and 95th percentiles, and values beyond these upper and lower bounds are considered outliers, marked with dots. Statistical differences between depths, if present, as inferred from a Kruskal-Wallis test and post-hoc Dunns test are noted by "ж" (Dunns test, $p<0.05$), "ж ж" (Dunns test, $p<0.01$) or "ж ж ж" (Dunns test, $p<0.001$).

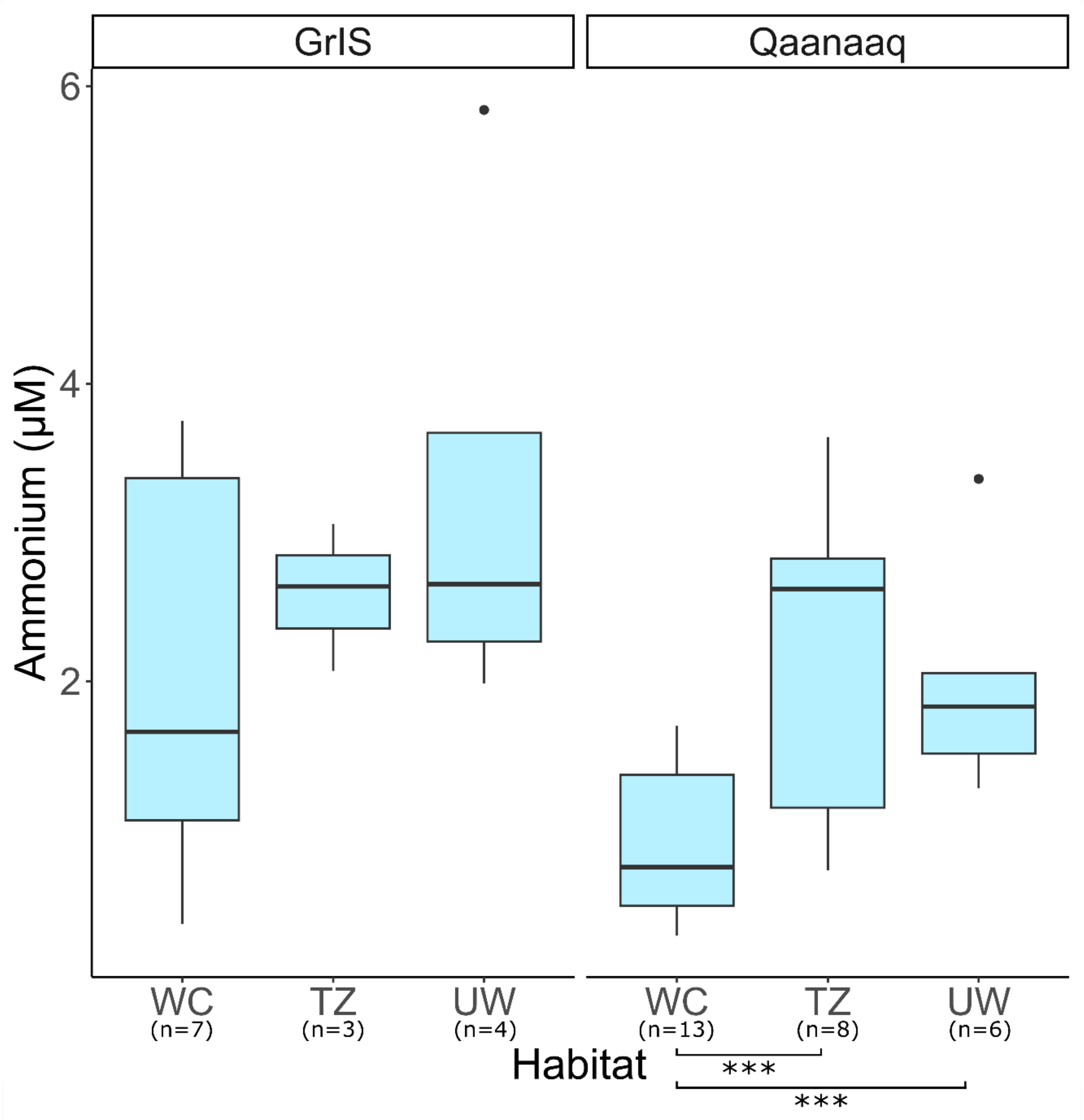

Figure S5. Boxplots showing the concentration of ammonium in solution for weathering crust (WC), transition zone (TZ) and unweathered ice (UW) samples collected at the Qaanaaq sampling locations and Greenland Ice Sheet (GrIS) sampling location. The number off replicates is indicated for each sample type in brackets. The line in the box denotes the median value, the box contains the 25th to 75th percentiles of dataset. The black whiskers mark the 5th and 95th percentiles, and values beyond these upper and lower bounds are considered outliers, marked with dots. Statistical differences between depths, if present, as inferred from a Kruskal-Wallis test and post-hoc Dunns test are noted by “ж” (Dunns test, $p<0.05$), “ж ж” (Dunns test, $p<0.01$) or “ж ж ж” (Dunns test, $p<0.001$).

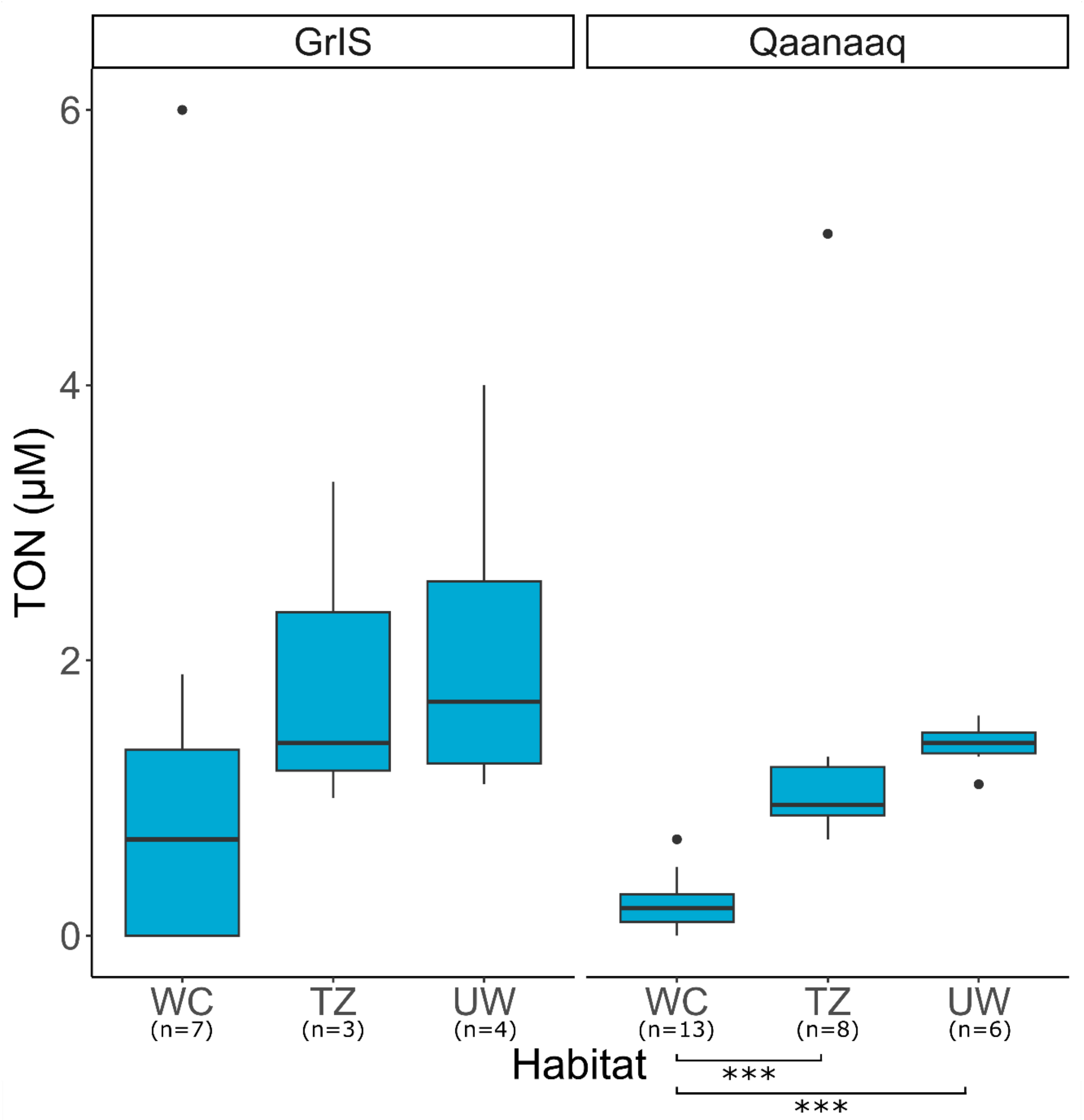

Figure S6. Boxplots showing the concentration of total oxidised nitrogen (TON) species (NO_3^- and NO_2^-) in solution for weathering crust (WC), transition zone (TZ) and unweathered ice (UW) samples collected at the Qaanaaq sampling locations and Greenland Ice Sheet (GrIS) sampling location. The number off replicates is indicated for each sample type in brackets. The line in the box denotes the median value, the box contains the 25th to 75th percentiles of dataset. The black whiskers mark the 5th and 95th percentiles, and values beyond these upper and lower bounds are considered outliers, marked with dots. Statistical differences between depths, if present, as inferred from a Kruskal-Wallis test and post-hoc Dunns test are noted by “ж” (Dunns test, $p<0.05$), “ж ж” (Dunns test, $p<0.01$) or “ж ж ж” (Dunns test, $p<0.001$).

References

1 Williamson, C. J. *et al.* Macro-Nutrient Stoichiometry of Glacier Algae From the Southwestern Margin of the Greenland Ice Sheet. *Frontiers in Plant Science* **12** (2021). https://doi.org/10.3389/fpls.2021.673614

2 Halbach, L. *et al.* Single-cell imaging reveals efficient nutrient uptake and growth of microalgae darkening the Greenland Ice Sheet. *Nature Communications* **16**, 1521 (2025). https://doi.org/10.1038/s41467-025-56664-6

3 Stevens, I. T. *et al.* [Pre-print] The formation and evolution of the supraglacial weathering crust on the Greenland Ice Sheet. *Journal of Glaciology* (2025).
